# On the Use of Ergotine in External and Internal Hemorrhages

**Published:** 1850-10

**Authors:** J. Bonjean

**Affiliations:** Chambery


*On the Use of Ergotine in External and Internal Hemorrhages.* By M. J. Bonjean, Chambery.—Ergotine when applied to wounds has the property, M. Bonjean states, of facilitating their cicatrization and moderating inflammation of the wounded tissues. Under its influence union takes place by the first intention, and cicatrization occurs without further assistance.

In certain cases ergotine may perform all the offices of the ligature. M. Bonjean enumerates the following circumstances attendant on a capital operation in which its employment was indicated:

1. When, in order to arrest a hemorrhage, it would be necessary to disturb the lips of a wound in which cicatrization is commencing.

2. When the patient manifests a tendency to gangrene of the cut surfaces.

3. When the source of the hemorrhage is from vessels imbedded in the inflamed and swollen tissues.

4. When the blood flows from many small arteries of which the orifices cannot be perceived.

5. When hemorrhage occurs from the sloughing of an eschar, as in gun-shot wounds, &c.

In these difficulties the application of ergotine is as often efficacious as the use of pressure is ineffectual. The application of ergotine supersedes ligature of the arteries, and effects cicatrization without interfering with the permeability of the artery.

The mode of employing ergotine is to dissolve it in five or six times its weight of water, for ordinary wounds; and in three or four parts, or even in a concentrated form, for more serious hemorrhages. A portion of the tow or lint is to be moistened with the fluid, and applied with gentle pressure to the surface previously wiped. When the hemorrhage does not return on the pressure being removed, another pledget moistened with the solution is to be laid over the former, and the limb bandaged as usual. Perfect rest is to be observed.

*Internal administration.*—Ergot of rye has been successfully employed:

1. As an excitant of uterine contractions.
2. As a stimulant to the muscular system in general.
3. In hemorrhages and certain fluxes.
4. In congestion of the uterus.
5. As a stimulant to the nervous system.

The latter poisonous effect of ergot of rye is due, according to M. Bonjean, entirely to its fixed oil. The preceding properties are due to the *ergotine* alone.

Simple extract, or ethereal tincture of ergot, both contain a portion of its poisonous principle. Pure ergotine is in the form of a solid extract of a deep brown color. In thin laminæ it presents a blood-red color. It has the odor of roast meat. Its taste is bitter. It is perfectly soluble in water, and this solution yields neither oil nor resin when heated with ether. (Gazette Méd.)—*Med. Gaz.*, *May* 1850, *p.* 787.

